# Quantitative trait loci for cell wall composition traits measured using near-infrared spectroscopy in the model C4 perennial grass *Panicum hallii*

**DOI:** 10.1186/s13068-018-1033-z

**Published:** 2018-02-03

**Authors:** Elizabeth R. Milano, Courtney E. Payne, Ed Wolfrum, John Lovell, Jerry Jenkins, Jeremy Schmutz, Thomas E. Juenger

**Affiliations:** 1 Department of Integrative Biology, The University of Texas at Austin, Austin, TX 78712 USA; 2 National Bioenergy Center, National Renewable Energy Laboratory, Golden, CO 80401 USA; 3 Department of Energy Joint Genome Institute, Walnut Creek, CA 94598 USA; 4 HudsonAlpha Institute for Biotechnology, Huntsville, AL 35806 USA

**Keywords:** *Panicum hallii*, Cell wall composition, QTL, NIRS, Lignocellulosic biomass, Bioenergy feedstock

## Abstract

**Background:**

Biofuels derived from lignocellulosic plant material are an important component of current renewable energy strategies. Improvement efforts in biofuel feedstock crops have been primarily focused on increasing biomass yield with less consideration for tissue quality or composition. Four primary components found in the plant cell wall contribute to the overall quality of plant tissue and conversion characteristics, cellulose and hemicellulose polysaccharides are the primary targets for fuel conversion, while lignin and ash provide structure and defense. We explore the genetic architecture of tissue characteristics using a quantitative trait loci (QTL) mapping approach in *Panicum hallii*, a model lignocellulosic grass system. Diversity in the mapping population was generated by crossing xeric and mesic varietals, comparative to northern upland and southern lowland ecotypes in switchgrass. We use near-infrared spectroscopy with a primary analytical method to create a *P. hallii* specific calibration model to quickly quantify cell wall components.

**Results:**

Ash, lignin, glucan, and xylan comprise 68% of total dry biomass in *P. hallii*: comparable to other feedstocks. We identified 14 QTL and one epistatic interaction across these four cell wall traits and found almost half of the QTL to localize to a single linkage group.

**Conclusions:**

*Panicum hallii* serves as the genomic model for its close relative and emerging biofuel crop, switchgrass (*P. virgatum*). We used high throughput phenotyping to map genomic regions that impact natural variation in leaf tissue composition. Understanding the genetic architecture of tissue traits in a tractable model grass system will lead to a better understanding of cell wall structure as well as provide genomic resources for bioenergy crop breeding programs.

**Electronic supplementary material:**

The online version of this article (10.1186/s13068-018-1033-z) contains supplementary material, which is available to authorized users.

## Background

Second-generation biofuels such as ethanol, butanol, and hydrocarbons are derived from vegetative lignocellulosic plant material [1–3] and are a critical component for current renewable energy strategies. These biofuels are advantageous over the current first-generation grain-based biofuels, because they use whole plant biomass and can have reduced ecological impact on land and water resources [3–5]. Lignocellulosic feedstocks include perennial prairie grasses such as switchgrass and big bluestem, tropical grasses such as *Miscanthus* and *Sorghum*, hardwoods such as poplar, and agricultural residues such as corn stover and sugarcane bagasse. These feedstocks have the potential to generate two to three times more biomass than first-generation grain-based feedstocks [6, 7] annually on marginal or non-agriculture land, or as secondary agricultural products. Current second-generation fuel conversion methods estimate 70–90% recovery of glucose and other soluble carbohydrates necessary for bioethanol and other types of biofuel conversions from these feedstocks [8, 9].

Historic improvement efforts in lignocellulosic biofuels have been primarily focused on increasing biomass feedstock production yield [4, 7, 10]. Decades of forage research has found that the quality of feedstock can affect the digestibility of forage in rumen guts [11] and can lead to increases in milk, fiber and biofuel conversion yields [12–15]. Feedstock quality for lignocellulosic plants is dependent on the composition of the cell wall. The fuel precursor carbohydrates in lignocellulosic feedstocks are bound in crystalized polysaccharide polymers and interwoven with a lignin matrix that provides both structure to the plant and protection from herbivores and pathogens [16, 17]. High-quality biofuel feedstocks have large quantities of accessible carbohydrates while maintaining structural integrity and defense mechanisms in the field.

Cellulose, hemicellulose, and lignin are the three main components of the cell wall in lignocellulosic plants [18]. Cellulose is a polymer of β linked d-glucose units. Hemicellulose is a polysaccharide composed of a mix of 5- and 6-carbon monosaccharides with the primary component in monocotyledons being 5-carbon xylan [8, 19]. Crystalline cellulose and hemicellulose molecules are intertwined with a phenylpropanoid polymer lignin matrix and provide both structural support and protection against natural enemies [17].

Biofuel conversion technologies are in a state of continuous development and improvement, but typically begin with a combination of mechanical, chemical, or thermal stresses. Pretreatment is followed by saccharification and fermentation, either sequentially or simultaneously [20, 21]. Independent of the specific method used, all biofuel conversion processes will benefit from well-defined plant tissue characteristics. Phenotypic and genotypic characterization of cell wall components and their interaction with agronomic growing conditions in the field will contribute to quality biomass production.

Plant tissue characterization in forage crops has been historically well-studied in the field of agronomy and is based on a number of longstanding methods. However, some popular methods can be inaccurate or impractical for large-scale studies. The long used detergent analysis method only provides a coarse quantification of cell wall components and has many known biases for lignocellulosic tissue [22–24]. Current procedures based on the Uppsala method [25] are more accurate but can be time- and cost-prohibitive for large sample studies. Near-infrared spectroscopy (NIRS) paired with multivariate analysis can be a quick, and non-invasive method for studying cell wall components. Primary analytical data and near-infrared (NIR) spectral data are used to build a multivariate predictive model that can then be used to predict composition based on spectral data from a sample of unknown composition. In this work, we use established laboratory analytical procedures (LAPs) based on an updated Uppsala method [5] to generate primary compositional analysis data for a subset of ‘calibration samples’. We then collect NIR spectral data from the calibration dataset, and use multivariate analysis to build a predictive model that can be applied to a larger spectral dataset of samples. NIRS has been used for a variety of agricultural applications from estimating seed fat content to green tea leaf alkaloids [26]. In biofuels, NIRS has been used to characterize cell wall components of switchgrass [27], corn stover [28], *Miscanthus* [29], *Sorghum* [30, 31], mixed grasses [32], and mixed wood [33] among others. Calibration models are most accurate when used to predict strict tissue composition [32] but can also include derived components such as total carbohydrate release [28, 30] and theoretical ethanol yields [27]. Several important applications have resulted from using NIRS for rapid analysis of cell wall traits. It is now convenient to assess biomass quality upon arrival at a biorefinery and quantify quality differences across environments, as water and other abiotic factors are known to have a large impact on yield and other biomass traits [34].

Understanding the genetics of cell wall components will lead to a better understanding of cell wall recalcitrance [10] as well as aid the generation of high-quality feedstock. The genetic architecture of economically relevant traits is important for locating large effect functional variants in the genome and for understanding how a quantitative trait, like tissue composition, will respond to selection in breeding programs. Genetic mapping of quantitative trait loci (QTL) is the first step in locating large effect variants and determining the genetic architecture of a trait and in implementing marker-assisted selection in breeding programs. Thus far, genetic analysis of cell wall traits using NIRS is limited to two studies in corn stover [28, 35]. To date, the authors are only aware of one published study that maps QTL for tissue characteristics as predicted by NIRS. In that study, Lorenzana et al. [28] find significant genetic variation, moderate heritability, and many QTL with small effects. There are a number of QTL studies for tissue characterization in forage crops based on the detergent system of analysis [36]. However, results from these analyses are known to underestimate lignin and bias cellulose and hemicellulose estimates due to incomplete solubilization of protein and other inhibitory elements, such as phenolic compounds, furans, and weak acids, in lignocellulosic tissue [22]. Whole genome surveys like QTL mapping and genome wide association studies (GWAS) have short-term benefits for marker-assisted breeding programs and long-term benefits in fine mapping, characterization of genetic architecture, and the ultimate discovery of genes involved in important biological processes.

**Fig. 1:**
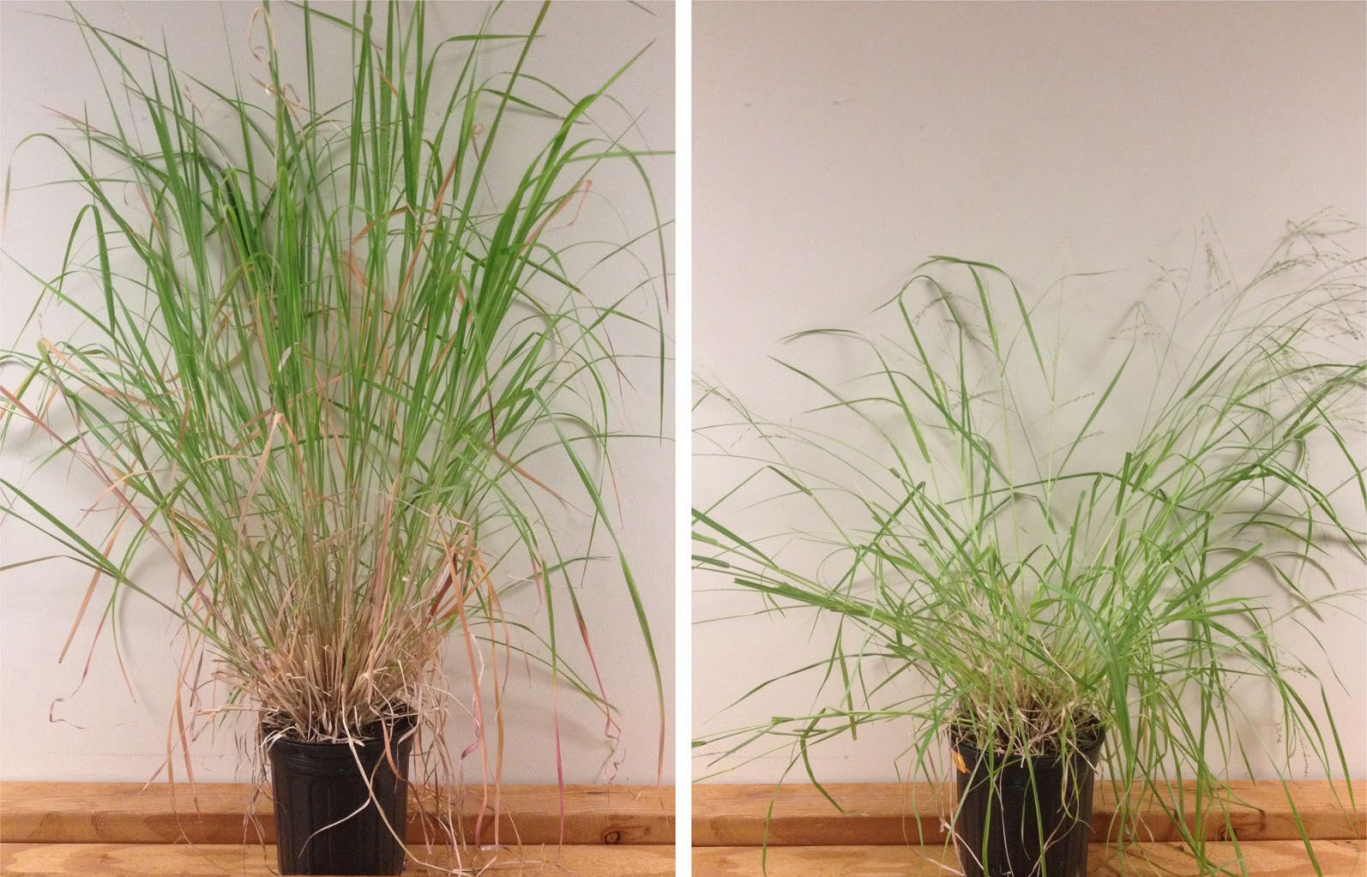
*Panicum hallii* var. *filipes* and var. *hallii*. *P. hallii* var. *filipes* (left) and *P. hallii* var. *hallii* (right) growing in 1-gallon containers

Hall’s panic grass (*Panicum hallii* Vasey) is an important genetic model system for C4 perennial grasses and for lignocellulosic biofuel crops in general [37–39]. *P. hallii* has a small (~ 550 Mbp) diploid genome, small stature, short generation time, and is self-compatible [39]. *P. hallii* has two distinct varieties, var*. hallii* found in xeric (dry) habitats and var*. filipes* found in mesic (moderate moisture) habitats (Fig. 1). The varietal distinction is similar to northern upland and southern lowland ecotypes in switchgrass [38], making *P. hallii* a good ecological as well as genetic model system. Current genomic tools include an annotated reference genome (http://phytozome.jgi.doe.gov/), transcriptome datasets, and a number of genetic mapping resources [37–39]. *P. hallii* shared a common ancestor with *Panicum virgatum* approximately 5 million years ago [40]. This phylogenetic proximity allows for close synteny and genomic resources, while the small tractable genome and self-compatibility are useful for functional assays and laboratory functional genomic experiments.

The purpose of this study is to explore the genetic architecture of tissue characteristics in a biofuel feedstock model grass. We do so using a QTL mapping approach in *P. hallii*. We use primary analytical methods of analyses for composition of reference samples and NIRS to build a *P. hallii* specific calibration model and quantify the cell wall composition and phenotypic correlations of tissue characteristics in an F_2_ mapping population between mesic and xeric varieties. We compare the cell wall composition of calibration samples to other lignocellulosic feedstocks to evaluate *P. hallii* as a model lignocellulosic grass. We estimate the number and effect size of QTL underlying cell wall components. Both the *P. hallii* specific NIRS calibration model and exploration into the genetics of lignocellulosic cell wall traits provide valuable resources for crop improvement in bioenergy grasses and further investigation into cell wall recalcitrance.

## Results

### Cell wall compositional analysis of calibration samples

Our study involved both laboratory compositional analysis and near-infrared spectral data analysis of a subset of samples to build a predictive model for cell wall composition. This model was then used to predict composition based on spectral data for the entire mapping population. We first report a full compositional analysis of the cell wall components for the 113 calibration samples in Table 1. Further details regarding the development of the predictive model are included as an additional file (Additional file 1). As expected, the *P. hallii* cell wall is composed primarily of lignin, glucan and xylan. We report 7.3% acetyl content in the calibration samples. Soluble sugars such as sucrose, glucose, and fructose are found in trace amounts and comprise 3.3% of the total biomass. Ash includes structural and non-structural inorganic compounds such as silica, potassium, calcium, sulfur, and chlorine and comprises 7.3% of the biomass.

**Table 1:** Composition of calibration samples

Composition component	Mean	SE	Max.	Min.
Extractives				
% Sucrose	1.6	0.21	6.3	0
% Soluble glucose	0.7	0.07	2.2	0.1
% Soluble fructose	1.0	0.11	5.2	0.1
% Water extractable others	10.3	0.26	13.7	6.3
% Ethanol extractives	3.8	0.06	4.8	3.2
Cell wall				
% Lignin	14.4	0.13	15.7	12.2
% Glucan	28.8	0.19	32.8	25.6
% Xylan	18.5	0.14	21.1	16.7
% Galactan	1.7	0.06	3.0	1.2
% Arabinan	3.7	0.07	5.4	2.6
% Acetyl	7.3	0.23	11.1	4.2
% Total ash	7.3	0.23	11.1	4.2
% Total protein	1.2	0.03	1.7	0.9
Total %	93.9	0.2	96.9	91.2

Composition statistics for 113 calibration samples reported as % of total dry biomass

*SE* standard error, *Max* maximum value, *Min* minimum value

**Fig. 2:**
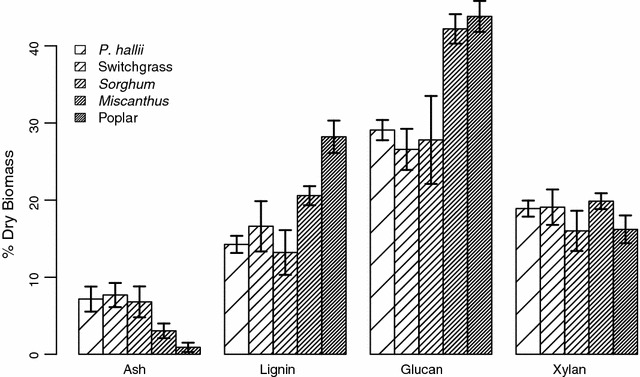
Comparison of major cell wall composition components in five lignocellulosic plant species. Mean and standard deviation values for NIRS calibration datasets are reported for *P. hallii*, switchgrass [27], *Sorghum* [30], *Miscanthus* [29], and poplar [41]

*Panicum hallii* has comparable cell wall composition to other perennial grass feedstocks. We evaluated the phenotypic similarity of *P. hallii* cell wall composition traits to three C4 grasses (switchgrass: [27]; *Sorghum*: [30]; *Miscanthus*: [29]) and one hard wood (poplar: [41]). We report the mean and standard deviation of percent dry biomass for ash, lignin, glucan, and xylan from NIRS calibration datasets with LAPs analogous to this study (Fig. 2; Additional file 2). We find that the composition of *P. hallii* cell walls is comparable to switchgrass and S*orghum* across all four traits and that relative xylan content is conserved across species (Fig. 2; Additional file 2).

### Parent means and phenotypic correlations for NIRS predicted traits

**Fig. 3:**
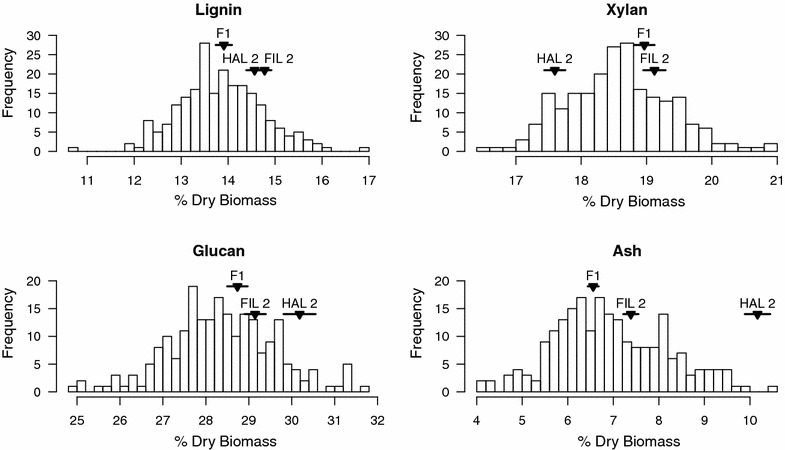
Phenotypic trait distributions for the F_2_ mapping population. Parent and F_1_ hybrid means are indicated by vertical arrows and standard error indicated by horizontal line

We found minimal parental divergence and significant phenotypic correlations for NIRS predicted cell wall traits. Model uncertainty was relatively low and similar to the uncertainty associated with primary methods of measurement, as indicated by the root mean square error of calibration (RMSEC) values for each trait (ash: 0.47, lignin: 0.46, glucan: 0.76, xylan: 0.67). All trait values are presented in units of percent total dry biomass. To assess patterns of parental divergence, we performed a *t* test of the difference between mean HAL2 and FIL2 trait predictions. Two of the four major cell wall components, ash and xylan, differed significantly between the parental lines (Table 2, Fig. 3). We tested for heterosis and found that the mean trait predictions for the F_1_ hybrid were significantly lower than both HAL2 and FIL2 for all traits except xylan (*p* < 0.05). There is some evidence for transgressive segregation, where the range of the F_2_ population exceeds that of the mean predictions for HAL2 and FIL2 (Fig. 3), in the traits without significant parental divergence, glucan and lignin. To explore patterns of trait correlations, we calculated phenotypic correlations among traits for parent lines and for the recombinant F_2_ progeny. The significance of correlations did not change across generations, so we only report the correlations for the mapping population. The three major cell wall structural components, glucan, xylan, and lignin are positively correlated. Ash is negatively correlated with lignin and xylan, and has no significant correlation with glucan (Table 3).

**Table 2:** Trait predictions for mapping population

	Ash	*N*	Lignin	*N*	Glucan	*N*	Xylan	*N*
F_2_	7.0 (0.08)	262	14.3 (0.04)	262	28.4 (0.07)	262	18.5 (0.04)	262
F_2_ range	3.0–11.4	–	12.7–16.6	–	25.3–31.8	–	17.0–20.9	–
F_1_	6.7 (0.11)	25	14.3 (0.09)	25	28.3 (0.17)	25	18.6 (0.09)	25
FIL2	7.2 (0.13)	25	15.0 (0.13)	25	29.0 (0.21)	25	18.8 (0.11)	25
HAL2	10.1 (0.25)	13	14.8 (0.16)	13	29.3 (0.23)	13	17.7 (0.13)	13
*p*val	< 0.0001	–	0.405	–	0.411	–	< 0.0001	–

Mean (SE) for cell wall trait predictions. *N* is the number of replicates for the parental lines and the number of F_2_ individuals measured for each trait, *p*val significance is the result of a *t* test for difference between the parental (HAL2 and FIL2) lines. Trait values presented as % dry biomass

**Table 3:** Phenotypic trait correlations for F_2_ population

Trait 1	Trait 2	*r*	*p*val
Ash	Lignin	− 0.23	< 0.0001
Ash	Glucan	0.07	0.24
Ash	Xylan	− 0.54	< 0.0001
Lignin	Glucan	0.54	< 0.0001
Lignin	Xylan	0.53	< 0.0001
Glucan	Xylan	0.50	< 0.0001

Pairwise Pearson product-moment correlation (*r*) and significance (*p*val)

### QTL analysis

**Fig. 4:**
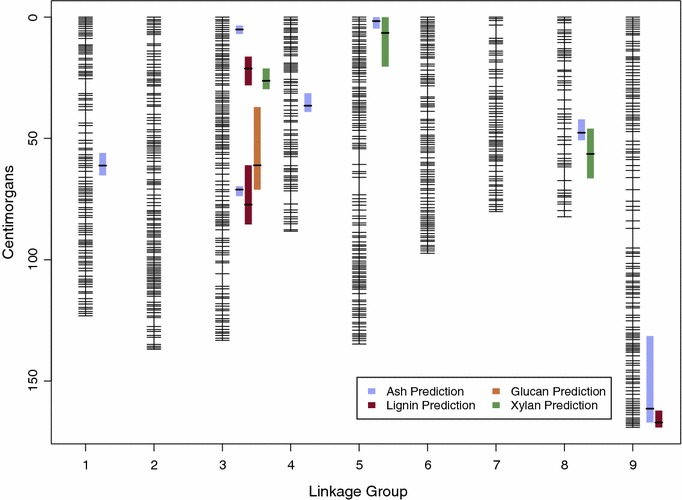
*Panicum hallii* genetic linkage map with QTL for cell wall traits. QTL plotted to the left of respective linkage groups. Color bars represent 1.5-LOD interval and horizontal line indicates location of QTL

We developed a new dense genetic linkage map for *P. hallii* based on expression polymorphisms derived from RNA-seq studies. This new map allowed an extensive study of genetic architecture for tissue characteristics using stepwise model building procedures in R/qtl. Fourteen QTL and one epistatic interaction were identified from four NIRS predicted cell wall traits using stepwise model selection (Fig. 4, Table 4). We found the most QTL for ash (totally 7) and detected at least one QTL for all the cell wall traits at an alpha of 0.1. Six QTL colocalize to linkage group (LG) 3 in addition to colocalizing QTL for pairs of traits on LGs 5, 8 and 9. Ash and xylan have a significant negative phenotypic correlation and have collocating QTL on both LGs 5 and 8. The allelic effects for these QTL are all in the expected direction of divergence, where the HAL2 allele increases ash content and decreases xylan content, at each location. We only find two occurrences where the allelic effects are not in the expected direction of parental divergence. These occur on LG 1 and LG 3 for ash. The QTL for ash on LG 3 have an epistatic interaction and contrasting allelic effects with substantial dominance.

**Table 4:** QTL and main effects for each cell wall trait

Trait	LG	Pos	1.5-LOD	LOD	*a* (SE)	*D* (SE)	PVE	PPD
Ash	1	61.17	56–65.2	7.73	− 0.449 (0.076)	− 0.026 (0.102)	5.55	− 31.23
Ash^a^	3	5.08	3.5–6.9	28.88	0.847 (0.091)	− 0.198 (0.119)	26.06	58.87
Ash^a^	3	71.11	69.7–73.8	22.17	− 0.932 (0.092)	− 0.145 (0.117)	18.57	− 64.79
Ash	4	36.48	31.4–39	5.06	0.134 (0.075)	0.493 (0.105)	3.53	9.33
Ash	5	1.63	0–4.7	9.15	0.563 (0.088)	− 0.136 (0.11)	6.67	39.16
Ash	8	47.65	42.1–50.7	13.09	0.629 (0.079)	0.132 (0.107)	9.95	43.74
Ash	9	161.45	131.5–167.1	6.57	0.379 (0.07)	− 0.092 (0.103)	4.66	26.31
Glucan	3	61.07	37–71.1	3.64	− 0.398 (0.097)	− 0.13 (0.136)	7.14	NA
Lignin	3	21.15	16.3–28.1	4.48	− 0.187 (0.054)	− 0.246 (0.074)	7.57	NA
Lignin	3	77.27	61.1–85.4	4.38	− 0.224 (0.056)	− 0.18 (0.074)	7.39	NA
Lignin	9	167.14	162.3–169.2	3.55	0.064 (0.051)	− 0.291 (0.074)	5.94	NA
Xylan	3	26.24	21.1–29.7	5.84	− 0.293 (0.059)	0.082 (0.078)	9.62	53.52
Xylan	5	6.52	0–20.4	4.80	− 0.275 (0.059)	0.049 (0.082)	7.82	50.26
Xylan	8	56.39	46–66.4	4.77	− 0.234 (0.058)	− 0.187 (0.077)	7.76	42.82

*LG* linkage group, *Pos* position of QTL on LG in cM, *1.5-LOD* 1.5 LOD drop confidence interval for each QTL in cM, *LOD* logarithm of odds score, *a (SE)* additive effect and standard error, *D (SE)* dominance deviation and standard error, *PVE* percent of additive variance explained by each QTL, *PPD* percent of parental divergence explained if applicable

^a^Epistatic interaction

The largest percent variance explained (PVE) by a single additive QTL is 10% for ash on LG 3. However, two additive ash QTL that also share an epistatic interaction, explain 18.6 and 26% of the variance. Total PVE, calculated using a full QTL model for each trait, is 67.5% for ash, 7.1% for glucan, 20.8% for lignin, and 23.0% for xylan. Additive effects range from 0.06 to 0.9% total biomass and dominance effects range from 0.03 to 0.5% total biomass. We calculated the mean difference between HAL2 and FIL2 for ash and xylan and found that the percent of parental divergence (PPD) explained by each loci ranges from 9.33 to 64.79% for ash and from 42.82 to 53.52% for xylan.

## Discussion

We found almost half of cell wall QTL localize to LG 3 in *P. hallii*. In addition, we discovered significant positive correlations between cellulose, hemicellulose, and lignin suggesting a potential for pleiotropic genetic architecture among these traits. This has implications for recalcitrance and the trade-off of diverting energy to sugar storage or building a structural matrix. Production of cellulose, hemicellulose, and lignin is sequential in the life stages of a plant. Cellulose is the first structure to form in new cell walls, then hemicellulose is added to the cellulose, joined with a series of covalent and non-covalent bonds, and lignin is the last component to be made. The hardening of a plant stem late in maturity is termed lignification. Understanding the timing and trade-offs in this process is important for biomass harvest, so as to avoid unnecessary hindrance to fuel conversion. Interestingly, using the same *P. hallii* F_2_ mapping population, Lowry et al. [38] localized 9 QTL to LG 3. These QTL include morphological traits such as tiller, leaf, and reproductive characteristics, and physiological traits such as CO_2_ assimilation rate, stomatal conductance. The density of functional alleles in this chromosome region suggests the possibility of a major developmental regulator controlling growth with concomitant impacts on important cell wall characteristics.

Ash content is a composite trait driven by the accumulation of inorganic molecules in plant tissues [42]. Primarily, high ash content reduces the overall density of energy convertible material in biomass. However, ash is inclusive of all structural and non-structural inorganic material and the type and function of inorganic molecules in plants are diverse. It is not surprising that we found the largest and most numerous QTL in our study associated with ash content. Wang et al. [43] found that switchgrass ash was composed of 67% SiO_2_, 12% CaO and many other mineral oxides in small concentrations. The high silica content can melt and fuse together when biomass is thermochemically pretreated [44], thereby causing problems when scaling up fuel conversion methods at the biorefinery. However, if harvested, the inorganic residue can be recycled into fertilizer [45] or a cement additive [43, 46]. Silica content has many functions in plant tissue including defense from abiotic and biotic stress [47, 48]. While there is mixed evidence regarding trade-offs in silica and carbon-based defenses [49, 50], silica has been found to reduce herbivory in both switchgrass and *Miscanthus* [51].

Hemicellulose is one of only three main structural components in the cell wall, therefore removal of any component would presumably lead to less structural integrity of stem tissue. However, there is tantalizing evidence that xylan, the primary structural pentose in monocotyledon hemicellulose, is linked to xylem production. Brown et al. [52] found that *Arabadopsis* xylan knockouts result in dwarfing and collapsed xylem. Reduced xylan in rice and corn leads to a droopy stature [53]. We found that parental HAL2 line contains, on average, 1.1 mg/g less xylan than FIL2 and no significant parental divergence between glucan or lignin. We also observe that *filipes* stems are more upright and erect in stature than *hallii* (Fig. 1).

Lignocellulosic feedstock species vary in cell wall quality and composition as well as in availability of genomic resources for crop improvement. This is only the second study to use NIRS for QTL mapping of cell wall traits. Many of the well-developed grass genome resources come from long domesticated crops such as corn, sugarcane, and sorghum. These crops have been bred for high-quality land use and intensive agricultural practices. Whereas perennial grasses such as switchgrass, *Miscanthus* and *Andropogon* are only recently being cultivated for agriculture and their genomes have not been subject to 8–10,000 years of anthropogenic selection. Variation in types and composition of lignocellulosic feedstocks is advantageous in that it broadens the range of acceptable habitats that can be used to produce substantial biomass material to meet global demand for renewable energy sources. However, diversity in the composition and quality of lignocellulosic feedstocks is disadvantageous for the biofuel conversion process, because biorefineries require uniform and high-quality biomass. Understanding the underlying genetic architecture of these quality biomass composition traits will lead to a better understanding of the structure and function of cell walls. We did not find significantly large effect QTL, but the loci we did detect account for two-thirds of the observed variation in ash and one-fifth of the observed variation in both lignin and xylan. Future studies that build on this research, including fine mapping and additional environments, will contribute to breeding efforts and crop development of bioenergy feedstocks.

## Conclusions

*Panicum hallii* serves as the genomic model for the emerging biofuel crop, switchgrass (*P. virgatum*). Near-infrared spectroscopy (NIRS) provides a rapid and economical approach for compositional analysis. We developed a new NIRS calibration model for *P. hallii* to quantify natural variation in tissue quality and discovered many clustered QTL underlying the genetic architecture of tissue quality in this emerging C4 perennial grass model system. Our model, and future explorations into the genetics of lignocellulosic cell wall traits, can provide valuable resources for crop improvement in bioenergy grasses and further investigation into cell wall composition.

## Methods

### Plant material and genetic map

The mapping population was generated by crossing single inbred accessions of two morphologically distinct varieties of *P. hallii*, where var*. hallii* (HAL2 genotype) was the dam and var*. filipes* (FIL2 genotype) was the sire. Both accessions were collected from wild populations in 2010 and the cross was made in 2011. A single self-pollinated F_1_ hybrid generated all the seeds for the F_2_ mapping population. The F_2_ progeny were grown under 16 h days in a glasshouse at the University of Texas at Austin in the fall of 2011. Details of greenhouse propagation as well as QTL for morphological and physiological traits are detailed in Lowry et al. [38]. A linkage map was generated by genotyping 264 F_2_ individuals. The map contains 3541 markers derived from RNA-seq based expression polymorphism data and spans 1045 cM with an intermarker map distance of ~ 0.5 cM. Details of linkage map construction can be found in supporting experimental procedures (Additional file 3). The linkage map itself and genotypes of the mapping populations are provided as supplemental data (Additional file 4).

Clonal replicates of the parental genotypes and their F_1_ hybrid, and individual F_2_ progeny were planted in the field in October 2012. The field experimental site was located in a prairie field (30.182° N, 97.879° W) at the south end of the Ladybird Johnson Wildflower Center (Austin, TX). Prior to planting, the field was covered with weed barrier cloth (Sunbelt 3.2 oz., Dewitt, Sikeston, MO, USA). For planting, holes were cut in the cloth and a mechanical auger was used to drill holes in the soil. Plants were arrayed into rows with 1.2 m spacing between rows and 40 cm spacing between plants, along rows. Due to exceptionally low rainfall in the fall of 2012, plants were watered as needed through November and early December to ensure establishment. Irrigation was ceased once plants entered winter dormancy. The experimental plants emerged from dormancy in the spring of 2013.

### NIRS phenotyping and model building

Plant tissue for NIRS analysis was harvested at the end of the growing season in 2013. Approximately 20–40 R3 stage tillers [54] were harvested from each of 262 F_2_, 13 HAL2, 25 FIL2, and 25 F_1_ plants. Tillers were dried to < 4% moisture at 50 **°**C and knife-milled to ≤ 2-mm particle size (Thomas Model 4 Wiley Mill, Thomas Scientific, Swedesboro, NJ, USA). Samples were homogenized by riffling (Gilson Spinning Riffler SP-230, Gilson Company Inc., Lewis Center, OH, USA) for uniform particle size distribution. A Thermo Antaris II Fourier Transform (FT)-NIR spectrophotometer (Thermo Scientific Inc., Madison, WI, USA) was used to scan each sample. The details of this process have been provided in Additional file 1 and are similar to the scanning procedures previously reported by Payne et al. [32] for high throughput scanning. A subset of 113 samples was chosen for model calibration based on their spectral distribution in multi-dimensional space. The sample selection and analytical methods used for the calibration dataset are detailed in supporting experimental procedures (Additional file 1) but outlined here for convenience. In summary, we performed a multi-step analytical procedure involving two-stage solvent extraction of the biomass samples (water then ethanol) followed by two-stage acid hydrolysis of the extracted biomass following the established protocol by Sluiter et al. [5]. We report the following values (all with units of % dry matter): water extractives, ethanol extractives, structural carbohydrates (glucan, xylan, galactan, arabinan), lignin (both acid soluble and acid-insoluble), and structural and non-structural inorganics, collectively termed “ash”. We also measured the sucrose, free glucose and free fructose concentration in the water extractives fraction. Structural carbohydrates are measured as soluble monomeric sugars using HPLC, and then converted to a structural (anhydro) basis.

We developed a preliminary partial-least-square (PLS-2) multivariate calibration model using near-infrared spectral data for the prediction of the most abundant cell wall components glucan, xylan, lignin, and ash. The model was fully cross-validated using the “leave-one-out” method, where a single sample is removed from the model, and the model rebuilt without the sample. Model uncertainty was approximated using RMSEC and the root-mean-square-error of the cross-validation (RMSECV). We then used the model to calculate a prediction for structural carbohydrates, lignin, and ash content for all remaining samples. Each sample prediction was associated with a measure of uncertainty or deviation from the mean predicted value. Any sample prediction, where twice its deviation was greater than or equal to the model’s RMSEC, was not used in the subsequent analysis. All laboratory procedures, NIRS analysis, and model development were performed at the National Renewable Energy Laboratory in Golden, CO. Phenotype data for the mapping population is provided as supplemental data (Additional file 5).

### QTL analysis

We mapped QTL for cell wall traits in the mapping population using a stepwise multiple-QTL model fitting method as implemented in the R package *R/qtl* [55]. All QTL scans were performed using a normal model and Haley–Knott regression based on a dense 0.5 cM grid of pseudomarkers generated using the calc.genoprob function. We calculated logarithmic odds-ratio (LOD) penalties for main effects and interactions for each trait through 1000 permutation of the scantwo function at an alpha of 0.1. The QTL significance threshold is relaxed, because this is an exploratory study in a new plant system and minimizing the number of false negative results is of greater importance than detecting false positive results. We conducted a forward/backward stepwise search for models with a maximum of 10 QTLs that optimized the penalized LOD score criterion. We calculated the 1.5 LOD drop interval of the QTLs in the best-fit models. We also used the best-fit stepwise model for each trait to calculate the additive effect, dominance deviation, and percent of variance explained (PVE) for each QTL using the makeqtl and fitqtl functions of *R/qtl*. We calculated the phenotypic difference between parental HAL2 and FIL2 lines and the percent of parental divergence (PPD) explained by the additive effect of each QTL for the traits with significant mean differences between parents.

## Additional files

**Additional file 1.** NIRS analysis and sample selection. Detailed methods regarding NIRS model calibration and analysis.

**Additional file 2.** Comparison of major cell wall composition components in five lignocellulosic plant species. Table of data to accompany Fig. 2.

**Additional file 3.** *Panicum hallii* genetic map construction. Detailed methods regarding linkage map construction from RNA-seq based expression polymorphism data.

**Additional file 4.** *R/qtl* genotype input file. File containing the *P. hallii* genetic linkage map and genotypes for the mapping population.

**Additional file 5.** *R/qtl* phenotype input file. File containing the NIRS phenotypes for the mapping population.
