# Supplementary material for: Quantitative trait loci for cell wall composition traits measured using near-infrared spectroscopy in the model C4 perennial grass Panicum hallii
Source: Biotechnol Biofuels. 2018 Feb 3;11:25. doi: 10.1186/s13068-018-1033-z (PMC5797396; doi:10.1186/s13068-018-1033-z)
Supplement: Supplementary file 1 — Additional file 1. NIRS analysis and sample selection. Detailed methods regarding NIRS model calibration and analysis. [file 13068_2018_1033_MOESM1_ESM.doc]

**Additional File 1**

*NIRS Analysis & Sample Selection*

A total of 325 *Panicum hallii* samples were prepared at the University of Texas at Austin (UTA) and scanned by the National Renewable Energy Lab (NREL) using a Thermo Antaris II FT-NIR spectrometer with Autosampler RS attachment (Thermo Scientific Inc., Madison, WI, USA). The autosampler attachment uses disposable 2 dram borosilicate glass vials for sample scanning. The resulting spectra were an average of 128 scans per sample using the wavenumber range of 12000 to 3300 with a resolution of 8 cm^-1^ (3.857 cm^-1^ data spacing). A subset of 113 samples from the 325 sample population was selected for wet chemical analysis. Bulk material for these samples was sent to NREL where it was subsampled for additional NIR scanning as described above. Spectra used to develop the final calibrations reported here were an average of spectra obtained from the original preparations from UTA and scanned by NREL, and bulk samples prepared by NREL and scanned by NREL.

The 113 samples for compositional analysis and calibration development were selected using an iterative process. All sample spectra were mathematically preprocessed using the standard-normal-variate (SNV) for scatter correction followed by Savitsky-Golay first derivative, second order polynomial with 21 point smoothing for baseline correction. The spectral range was then reduced, 4000 cm^-1^ to 7999 cm^-1^, to remove spectral noise due to increased variation in the signal response. Of the 325 samples available, samples with less than 15 grams of total material were immediately eliminated for compositional analysis so as not to significantly reduce or exhaust the physical sample. This eliminated 101 samples from consideration. Using only spectra from samples with sufficient quantities for wet chemical analysis, a principle component analysis (PCA) was preformed using Unscrambler X 10.3 (Camo USA) with weighted spectra (1/standard deviation) and full cross validation. Full cross-validation refers to the practice of validating a model by removing a single sample from the calibration set, rebuilding the model and predicting the removed sample no longer included in the model. An initial 50 samples were selected by applying the Kennard-Stone (KS) algorithm to the PCA across two principle components [1]. This algorithm selects a specified number of samples from a population based on spectral variation across a select number of principle components. An additional 12 samples were selected for calibration as spectral outliers. In this case, these samples were those which were furthest from the center of the space described by the PCA across two principle components (PCs).

Following compositional analysis of the 62 samples, an initial Partial Least Squares (PLS) calibration was developed (data not shown.) This preliminary model did not achieve the robust modeling statistics we desired; therefore a second set of samples was selected for compositional analysis to improve upon the preliminary 62 sample calibration. An additional 50 samples were selected using a similar process as detailed above using the KS algorithm applied to the 325 sample PCA. However, samples with quantities less than 10 g were excluded from selection (49), as well as the previously selected 62 samples for wet chemical analysis. One additional sample outlier was selected as previously described for a total of 51 additional samples for wet chemical analysis.

*Compositional Analysis*

All samples selected for compositional analysis were analyzed following the publically available Laboratory Analytical Procedures (LAPs) developed by NREL [2]. The history and uncertainties associated with these methods have been reported [3, 4]. Samples were dried to less than 5% moisture and sequentially extracted using water and ethanol for nonstructural component quantification which included: sucrose, free glucose, free fructose, water extractives, and ethanol extractives. This was followed by a two phase sulfuric acid hydrolysis for determination of structural components including lignin, glucan, xylan, galactan, arabinan, fructan and acetic acid. Samples were also analyzed for protein, starch, and ash content. All measured constituents were corrected to a dry weight basis.

*NIR Multivariate Calibration Development*

The first calibration developed in support of this work used all 113 samples analyzed by wet chemical methods. The purpose of this calibration was to fully capitalize on the range of constituent values provided by wet chemical analysis and for compositional prediction of all 325 panicum hallii samples. The spectra for the 113 samples were mathematically preprocessed and the spectral region reduced prior to model development as previously described. A Partial Least Square (PLS) multivariate calibration was developed using Unscrambler X 10.3 (Camo USA) software. A PLS-2 calibration was developed which relates multiple independent variables such as glucan, xylan, lignin, and ash to a function of the dependent variable or spectra. The model was fully cross validated using the “leave-one-out” method. The optimal number of factors for this model was determined using RMSEC and RMSECV values which closely approximated the uncertainties in the primary methods of measurement. The total explained variance in the data accounted for by the model was also used to determine the appropriate number of factors for calibration using the fewest number of factors possible. This included a consideration of the explained variance maxima (close to 100%) for validation as well as factors at which divergence of the calibration and validation variance began to occur. Outlier removal did not lead to significant improvements in calibration statistics therefore no outliers were removed with the intention that this variability would improve overall predictive performance.

A second calibration was developed by selecting 95 samples for calibration and 18 samples for validation. The purpose of this calibration was to adhere to more traditional methods of calibration development which use a set of samples for model development and a separate set of samples for validation of that model. The 95 samples for calibration were selected using the KS algorithm as previously described for selection of samples for chemical analysis. However, in this case the algorithm was applied to the 113 sample PLS-2 calibration described above across two PCs selecting 95 samples for use in calibration. The remaining 18 samples were designated for validation. As with the 113 sample calibration, removal of outliers did not significantly improve model statistics or its predictive ability, therefore no samples were excluded for this reason.

*Sample Prediction of Composition*

The 113 sample calibration was used for the prediction of glucan, xylan, lignin, and ash for the remaining 212 samples not analyzed by wet chemical methods. The 212 Panicum hallii sample spectra were preprocessed as previously described and then predicted on the 113 sample PLS2 calibration. Sample predictions of composition in UnscramblerX are accompanied with “Deviations” which are an estimate of prediction uncertainty. The quality of the prediction was assessed by comparison of the deviation of the prediction to the RMSEC of the calibration on which it was predicted. Samples with predicted deviations greater than twice the RMSEC of the calibration would be excluded from further use. In this case no samples predicted with deviations larger than twice the value of the RMSEC.

**Results and Discussion**

*NIR Multivariate Calibration and Sample Prediction*

A total of 113 samples were chosen for wet chemical analysis and subsequent calibration development which best represented the NIR spectral range of the population and therefore compositional range of the 325 *P. hallii* samples. Two calibration models were developed in support of this work based on these 113 samples. One calibration was developed as a proof-of-concept using 95 samples for calibration and 18 samples for validation. The other used all 113 samples for calibration and subsequent sample prediction with no validation set. The primary intent of calibration development for this work was to circumvent lengthy and labor intensive wet chemical analysis of all 325 samples associated with the reported experiment. The model’s primary purpose was to predict samples from the same population. Therefore, there was no specific interest in determining the 113 sample model’s ability to predict a truly external validation set to prove the robust nature of the final calibration. The authors are aware of the calibration’s current limitations but hope that it can serve as the foundation for a more robust *P. hallii* model in the future.

With development of the calibration/validation model, validation samples were not chosen based on spectral redundancy nor was a truly external validation set sought to test the “robust” nature of the final model. The validation set here is not external to the population and does not demonstrate the model’s ability to predict samples outside this specific experiment. The validation samples if anything are a training set. Several compositional constituents were available for model development, but glucan, xylan, and lignin as the most abundant cell wall materials were the focus of model development here. Ash was included for its implications on the bioconversion process. Descriptive statistics for the 95 calibration samples and 18 validation samples are provided in Table 1. Figure 1 provides an alternative view of these statistics with histograms presented for glucan, xylan, lignin, and ash. Summary statistics for the 95 sample PLS-2 calibration model are provided in Table 2. Included in this summary are values for RMSEC and RMSECV which reasonably approximate the uncertainties in the primary methods of measurement. The square of the correlation coefficient or coefficient of determination of the cross validation (R^2^) is also reported. These numbers are generally lower than the values for calibration but give a better sense of the models performance. Also included in this table are slope and intercept which describe the line of best fit for cross-validation. Summary statistics for the 18 sample validation set predicted on the 95 sample calibration are provided in Table 3. Figure 2 illustrates the predicted versus measured values for glucan, xylan, lignin, and ash for the calibration model. R^2^ values here are reasonable but lower (R^2^ < 0.80) for some constituents than a more mature model with a wider compositional range. Figure 3 illustrates the predicted versus measured values for the validation set, where predictions were obtained from the 95 sample calibration.

The final 113 sample calibration model was ultimately used to predict samples that were not analyzed by primary methods. Descriptive statistics of chemical composition for this sample set are provided in Table 4. Figure 4 also provides an alternative view of these statistics with histograms presented for the four constituents. Summary statistics for the PLS-2 calibration developed from this sample set are listed in Table 5. RMSECV values for this calibration are slightly higher than those reported for the 95 sample calibration but remain a reasonable approximation of the uncertainty in the primary methods of measurement. R^2^ values for cross-validation are slightly lower than those reported for the 95 sample calibration. Figure 5 illustrates the predicted versus measured values for glucan, xylan, lignin, and ash for the 113 sample calibration model. Again, R^2^ values are reasonable but lower (R^2^ < 0.80) than what would be expected for a more mature model with wider compositional range.

**References**

1. Kennard RW, Stone LA. Computer Aided Design of Experiments. *Technometrics* 1969, 11:137–148.

2. Standard Procedures for Biomass Compositional Analysis [http://www.nrel.gov/biomass/analytical_procedures.html]

3. Sluiter JB, Ruiz RO, Scarlata CJ, Sluiter AD, Templeton DW. Compositional analysis of lignocellulosic feedstocks. 1. Review and description of methods. *J Agric Food Chem* 2010, 58:9043–53.

4. Templeton DW, Scarlata CJ, Sluiter JB, Wolfrum EJ. Compositional analysis of lignocellulosic feedstocks. 2. Method uncertainties. *J Agric Food Chem* 2010, 58:9054–62.

**Tables**


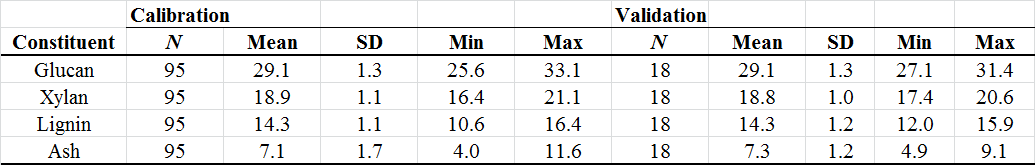


Table 1. Descriptive statistics of composition for the 95 calibration and 18 validation sample sets. Statistics for composition are reported on a percent dry weight basis (wt%). *N* number of samples, *SD* standard deviation, *Min* minimum value, *Max* maximum value.


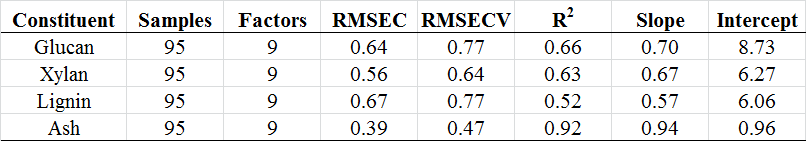


Table 2. Summary statistics for the PLS-2 95 sample calibration model for composition. RMSECV values are similar to the uncertainty in the primary analytical methods. Slope and intercept describe the line of best fit for cross-validation. *RMSEC* root-mean-square-error of the calibration model, *RMSECV* root-mean-square-error of the cross-validated model, *R^2^* square of the correlation coefficient of the cross-validated model.


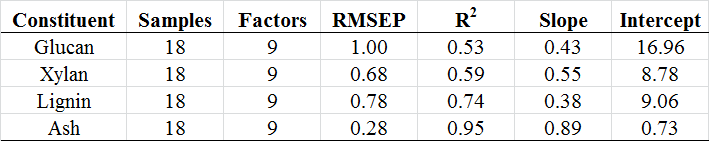


Table 3. Summary statistics for validation of the PLS-2 calibration model for composition. RMSEP values are similar to the uncertainty in the primary analytical methods. Slope and intercept describe the line of best fit for these samples. *RMSEP* root-mean-square-error of prediction, *R^2^* square of the correlation coefficient of the validation.


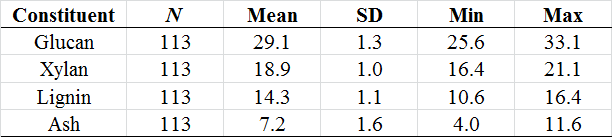


Table 4. Descriptive statistics of composition for the 113 calibration sample set. Statistics for composition are reported on a percent dry weight basis (wt%). *N* number of samples, *SD* standard deviation, *Min* minimum value, *Max* maximum value.


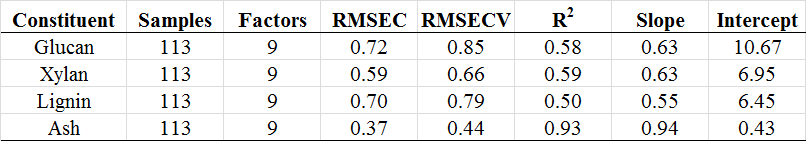


Table 5. Summary statistics for the PLS-2 113 sample calibration model for composition. RMSECV values are similar to the uncertainty in the primary analytical methods. Slope and intercept describe the line of best fit for cross-validation. *RMSEC* root-mean-square-error of the calibration model, *RMSECV* root-mean-square-error of the cross-validated model, *R^2^* square of the correlation coefficient of the cross-validated model.

**Figures**


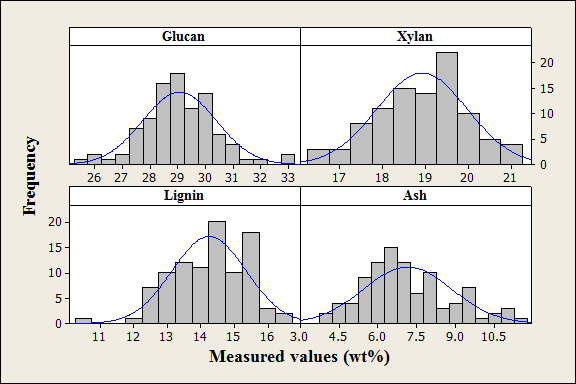


Figure 1. Histogram for glucan, xylan, lignin and ash for the 95 sample calibration set. Composition was measured on a percent dry weight basis (wt%). Frequency refers to the number of samples that fall into a given measured value range for each constituent. The blue line represents a normal distribution and is included for comparison to highlight any differences between the histogram and normality.


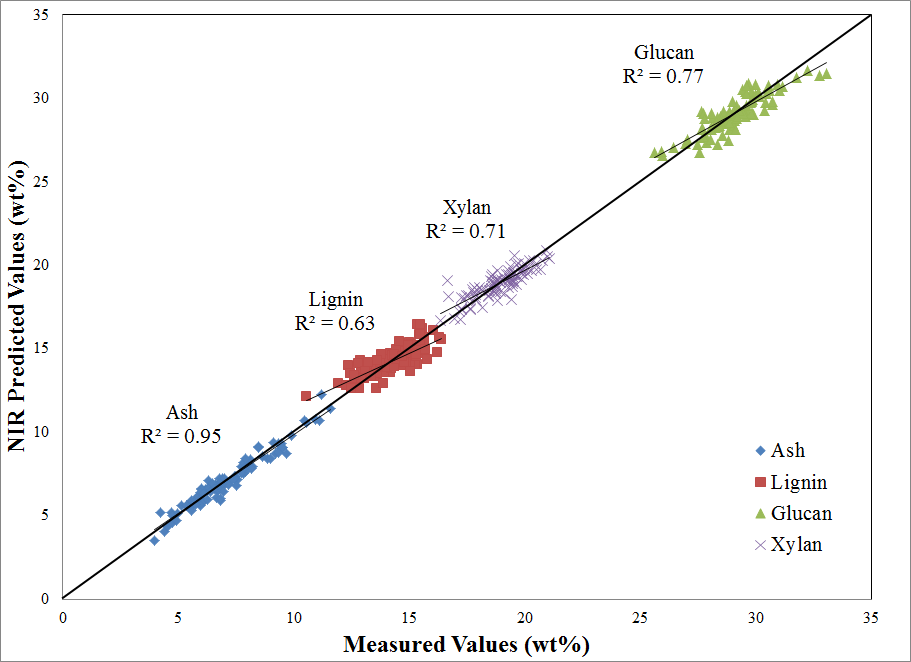


Figure 2. Predicted versus measured values of glucan, xylan, lignin, and ash for the 95 calibration samples. The x-axis represents the measured wet chemical values for composition on a percent dry weight (wt%) basis. The y-axis represents the NIR predicted values for composition from the 95 sample PLS-2 calibration equation.


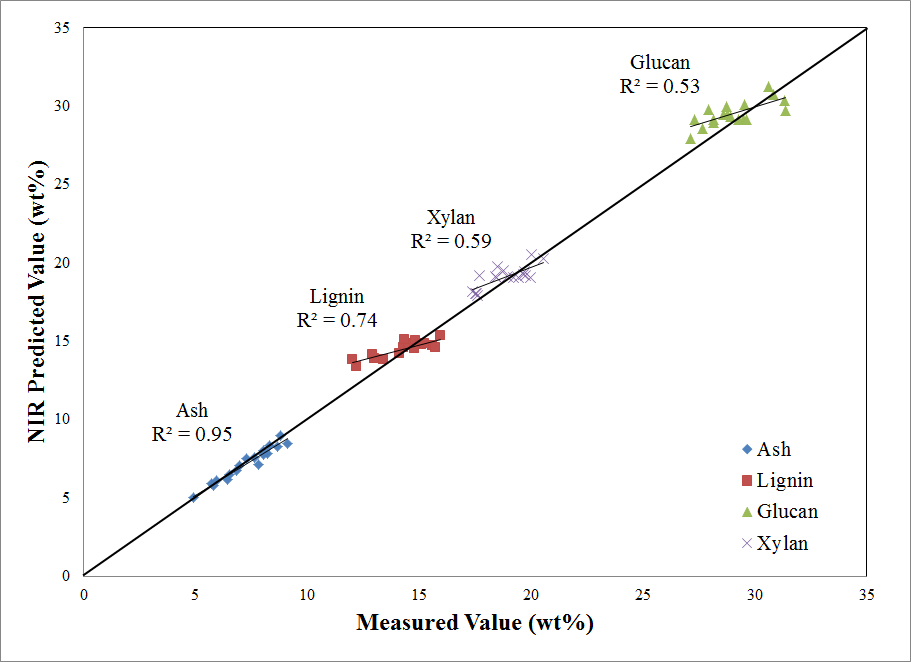


Figure 3. Predicted versus measured values of glucan, xylan, lignin, and ash for the 18 validation samples. The x-axis represents the measured wet chemical values for composition on a percent dry weight (wt%) basis. The y-axis represents the NIR predicted values for composition of the 18 validation samples predicted on the 95 sample PLS-2 calibration equation. The validation samples were not used to build the calibration model on which they were predicted.


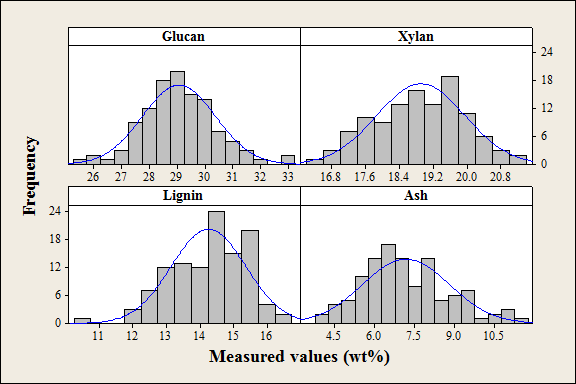


Figure 4. Histogram for glucan, xylan, lignin and ash for the 113 sample calibration set. Composition was measured on a percent dry weight basis (wt%). Frequency refers to the number of samples that fall into a given measured value range for each constituent. The blue line represents a normal distribution and is included for comparison to highlight any differences between the histogram and normality.


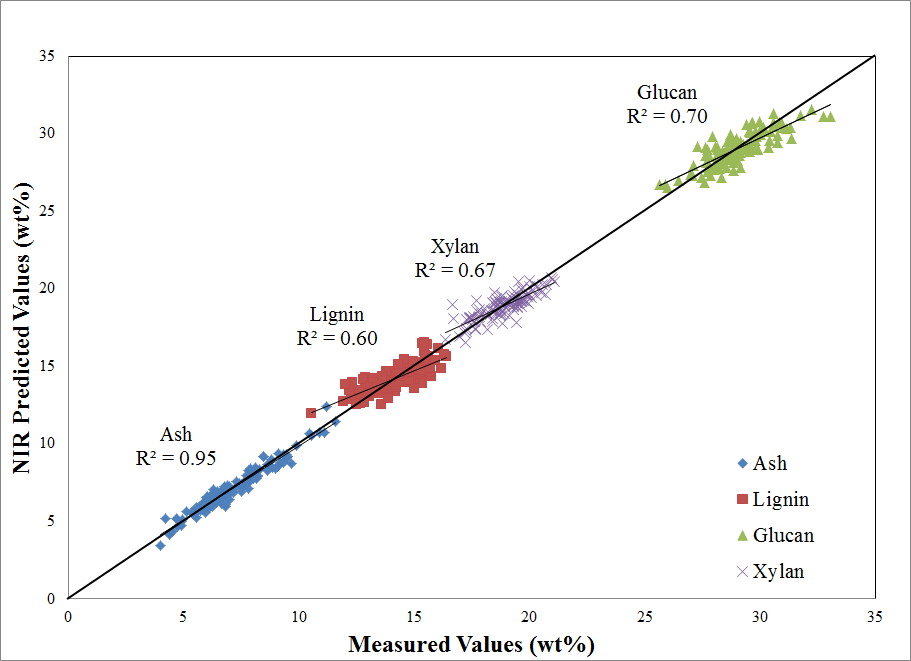


Figure 5. Predicted versus measured values of glucan, xylan, lignin, and ash for the 113 calibration samples. The x-axis represents the measured wet chemical values for composition on a percent dry weight (wt%) basis. The y-axis represents the NIR predicted values for composition from the 113 sample PLS-2 calibration equation.
