# Supplementary material for: Quantitative trait loci for cell wall composition traits measured using near-infrared spectroscopy in the model C4 perennial grass Panicum hallii
Source: Biotechnol Biofuels. 2018 Feb 3;11:25. doi: 10.1186/s13068-018-1033-z (PMC5797396; doi:10.1186/s13068-018-1033-z)
Supplement: Supplementary file 2 — Additional file 2. Comparison of major cell wall composition components in five lignocellulosic plant species. Table of data to accompany Fig. 2. [file 13068_2018_1033_MOESM2_ESM.doc]

**Additional File 2**

**Table S1.** Comparison of major cell wall composition components in five lignocellulosic plant species.

| **Composition Component** | ***P. hallii*** | **Switchgrass^a^** | ***Sorghum*^b^** | ***Miscanthus*^c^** | **Poplar^d^** |
| --- | --- | --- | --- | --- | --- |
| % Ash | 7.3 (1.6) | 7.69 (1.57) | 6.8 (2.0) | 3.05 (0.95) | 0.9 (0.6) |
| % Lignin | 14.3 (0.92) | 16.6 (3.26) | 13.2 (2.9) | 20.57 (1.24) | 28.2 (2.1) |
| % Glucan | 28.8 (1.33) | 26.57 (2.67) | 27.8 (5.7) | 42.19 (1.9) | 43.8 (2.0) |
| % Xylan | 18.5 (0.98) | 19.08 (2.29) | 16 (2.6) | 19.85 (1.03) | 16.2 (1.8) |
| n | 50 | 112 | 155 | 172 | 14 |

**Table S1.** Comparison of major cell wall composition components in five lignocellulosic plant species. Mean and standard deviation values for NIRS calibration datasets are reported as % dry biomass, number of samples included in each calibration set (n).

^a^Vogel et al. 2011

^b^Wolfrum et al. 2013

^c^Haffner et al. 2013

^d^Maranan and Laborie 2008
