# Supplementary material for: Quantitative trait loci for cell wall composition traits measured using near-infrared spectroscopy in the model C4 perennial grass Panicum hallii
Source: Biotechnol Biofuels. 2018 Feb 3;11:25. doi: 10.1186/s13068-018-1033-z (PMC5797396; doi:10.1186/s13068-018-1033-z)
Supplement: Supplementary file 3 — Additional file 3. Panicum hallii genetic map construction. Detailed methods regarding linkage map construction from RNA-seq based expression polymorphism data. [file 13068_2018_1033_MOESM3_ESM.doc]

**Additional File 3**

*P. hallii Genetic Map construction*

Total RNA was extracted from the Hal2 and Fil2 parents, the F1 hybrid and all F2 genotypes and sequenced at the Joint Genome Institute. Allele specific expression (ASE) counts were calculated across >16k genes and all genotypes following Lovell et al. [1]. These counts were TMM normalized in the R package *edgeR* to correct for differential sequencing depth and mapping biases.

ASE normalized counts were examined in the parental and F1 genotypes. To define the genes that would provide informative, co-dominant markers in the F2, we set three conditions: 1) mean counts for Fil-ASE must be >50 in Fil2 and <1 in Hal2 (10,099 genes), 2) mean counts for Hal2 ASE must be >50 in Hal2 and <1 in Fil2 (10,235 genes), and 3) median counts of both Hal2 and Fil2 ASE must be >10 (13,802 genes). The intersection of these three conditions resulted in 8,477 genes.

We called genotypes for each of the 8477 genes that satisfied our ASE criteria using Bayesian model-based clustering in the *mclust* R package [2,3] implemented in a custom R function genobyMclust (https://github.com/jtlovell/eqtlanalysis/blob/master/genobyMclust.R). This function concatenated the bivariate distribution of Hal2 and Fil2-specific counts then clustered each individual into one of three groups, simultaneously assigning a P-value for the strength of clustering for each individual. The three clusters were then assigned to the Fil2 (aa), Hal2 (bb), or heterozygote (ab) genotype. The genotype for each F2 was then output with a paired P-value representing the uncertainty of assignment to that cluster. Assignments with uncertainties P > 1e-10 were coded as missing. We culled this dataset to include only genes and individuals with < 5% missing genotypes and segregation distortion χ^2^ < 100. This resulted in 264 individuals and 3541 genes/markers.

Linkage groups and marker order were inferred via recombination fractions and maximum likelihood respectively, using default settings in JoinMap4. After examination of the marker order, it was clear that 17 individuals were experiencing a much higher rate of recombination than the other lines, an indicator of contamination or poor sequencing quality. While the initial map exceeded 6000cM, when these 17 individuals were excluded, the map shrank to just over 1000cM. These 17 erroneous individuals were excluded for all subsequent analyses.

The map constructed in JoinMap4.1 was further processed in the R package *R/qtl* [4]. At each step of processing in *R/qtl*, the genetic map was re-estimated using the Kosambi map function and the Viterbi algorithm via the quickEst function of the R package *ASMap* [5], which utilized *R/qtl*’s internal functions to more efficiently estimate large maps. Markers separated by < 0.1cM were culled to the best single marker using the dynamic programming algorithm [6], weighted equally by the proportion of missing data and degree of segregation distortion. Marker culling was implemented in *R/qtl*’s pickMarkerSubset function. Duplicated markers we also dropped.

One large rearrangement of markers was required on chromosome 2, where recombination fraction patterns indicated inverted marker order. Marker order was then further refined using the ripple algorithm counting cross-over events within 5-marker windows. The marker order that required the fewest obligate cross-over event was retained for each chromosome. The final map was generated by re-running pickMarkerSubset retaining a single marker for each 0.5cM window. Finally, linkage groups were oriented by the order of markers in the *P. hallii* v1.1 annotation. For chromosomes 6-9, where orientation could not be determined from the annotation, linkage groups were oriented so that the inferred centromere was nearest to the proximate telomere.

**References**

1. Lovell JT, Schwartz S, Lowry DB, Shakirov EV, Bonnette JE, Weng X, Wang M, Johnson J, Sreedasyam A, Plott C, Jenkins J, Schmutz J, Juenger TJ. Drought responsive gene expression regulatory divergence between upland and lowland ecotypes of a perennial C4 grass. Genome Res. 2016;26:510-518.

2. Fraley C, Raftery AE, Murphy TB, Scrucca L. mclust Version 4 for R: Normal Mixture Modeling for Model-Based Clustering, Classification, and Density Estimation. Technical Report No. 597. Department of Statistics: University of Washington; 2012.

3. Fraley C, Raftery AE. Model-based clustering, discriminant analysis, and density estimation. Journal of the American Statistical Association. 2002;97:611-631.

4. Broman KW, Wu H, Sen Ś, Churchill GA. R/qtl: QTL mapping in experimental crosses. Bioinformatics. 2003;19:889–890

5. Taylor J, Butler D. ASMap: Linkage Map Construction using the MSTmap Algorithm. 2015. R package version 0.3-3.

6. Broman KW, Weber JL. Method for constructing confidently ordered linkage maps. Genet. Epidemiol. 1999;16:337–343.
